# Drivers of care and outcomes for people facing fetal conditions in the United States: a conceptual framework

**DOI:** 10.1038/s41372-025-02340-y

**Published:** 2025-07-02

**Authors:** Abigail B. Wilpers, Scott A. Lorch

**Affiliations:** 1 Department of Family and Community Health, University of Pennsylvania School of Nursing, Philadelphia, PA USA; 2 Research Institute, Children’s Hospital of Philadelphia, Philadelphia, PA USA; 3 Children’s Hospital of Philadelphia, Division of Neonatology, Philadelphia, PA USA; 4 Perelman School of Medicine, University of Pennsylvania, Philadelphia, PA USA

**Keywords:** Health care, Scientific community

## Abstract

Congenital anomalies, affecting 3–5% of pregnancies annually in the United States, are a leading cause of fetal and infant mortality. Despite advancements in fetal care, disparities in care access, quality, and outcomes persist and remain poorly understood. This perspective introduces the Fetal Condition Care and Outcomes (FCCO) Framework, a conceptual model that integrates contextual, individual, structural, and process-level factors influencing care and outcomes. We build on prior adaptations of *Andersen’s Behavioral Model of Health Service Use* and the *Donabedian Structure, Process, and Outcomes Model*, expanding their application from risk-appropriate neonatal care to individuals’ whose pregnancies are complicated by severe fetal conditions. By synthesizing evidence across disciplines, we highlight critical gaps in understanding the drivers of disparities, including barriers to timely diagnosis, variations in counseling practices, and inequities in access to specialized services. This article calls for interdisciplinary research to ensure risk-appropriate, person-centered care for this high-risk population.

## Introduction

Congenital anomalies affect 3–5% of pregnancies annually in the United States and are a leading cause of fetal death and infant mortality [1]. These conditions, which include structural abnormalities like congenital heart disease (CHD) and genetic syndromes such as Trisomy 13, often require high-risk specialized prenatal and neonatal care [1]. A growing body of evidence highlights significant disparities in care and outcomes for individuals with fetal conditions, with socioeconomically disadvantaged groups experiencing delays in prenatal diagnosis of CHD and Black populations facing higher mortality rates among children with conditions like spina bifida [2–4]. Research on disparities in this area has been fragmented, often narrowly focused on specific aspects of care—such as prenatal screening or neonatal services—or isolated indicators of social determinants like socioeconomic status or race [5, 6]. Most studies have concentrated on congenital heart disease (CHD), leaving other fetal conditions underexplored [5]. These approaches cannot capture complex interactions that shape access, use, and care quality across the continuum of care that determine the outcomes for individuals facing any severe fetal condition [7]. A comprehensive conceptual model that integrates these components, mapping evidence onto a unified framework, is essential to understand the drivers of disparities within this population.

To address this need, we build on prior adaptations of *Andersen’s Behavioral Model of Health Service Use* and the *Donabedian Structure, Process, and Outcomes Model*, expanding their application from risk-appropriate neonatal care to encompass prenatal care for individuals’ whose pregnancies are complicated by severe fetal conditions [8, 9]. This perspective synthesizes key evidence, identifies critical gaps, and proposes future research directions that support pathways toward health equity in this high-risk population.

## Health services access and use in the context of fetal conditions

**Fig. 1:**
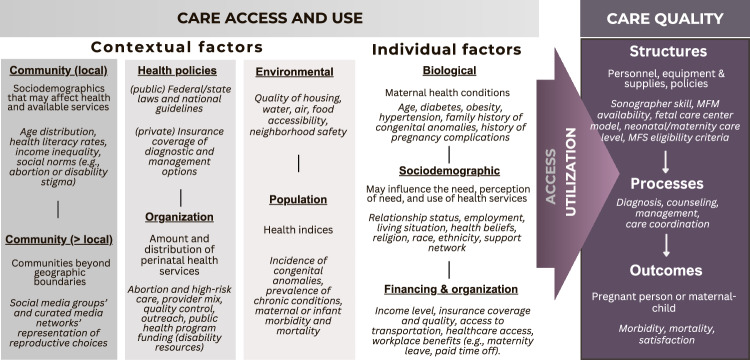
The fetal condition care and outcomes framework builds on and modifies the *Andersen’s Behavioral Model of Health Service Use* and the *Donabedian Structure, Process, and Outcomes Model.* The framework integrates contextual, individual, structural, and process-level factors that can influence care and outcomes.

**Fig. 2:**
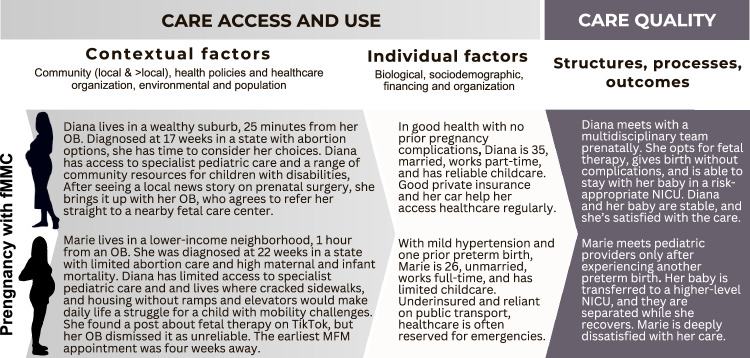
This figure illustrates the fetal condition care and outcomes model through case exemplars. These are fictionalized scenarios based on existing evidence and clinical experience, intended to ground the framework in real-world contexts.

Access to and use of health services by individuals with pregnancies affected by fetal conditions are influenced by a combination of contextual and individual characteristics. This approach is rooted in Andersen’s *Behavioral Model of Health Services Use*, which explains and predicts healthcare utilization [8]. This framework has been widely applied in healthcare, including the 2021 adaptation by Lorch et al., which illustrated how patient, community, and policy factors interact to influence access to risk-appropriate services for high-risk infants [10]. Building on these concepts, The Fetal Condition Care and Outcomes Framework (Fig. 1) examines the interplay of contextual and individual factors to determine both the perceived need for care and the ability to navigate healthcare systems. Together, these elements and two case examples (Fig. 2) provide a foundation for understanding disparities in care access and use within the context of fetal conditions.

### Contextual characteristics

#### Community factors

Structural and social factors within an individual’s local community significantly influence healthcare utilization, including perinatal care [11]. Communities with higher socioeconomic status (SES) and a large proportion of reproductive-age families often have better perinatal resources, while economically declining areas with youth outmigration face reduced access [11]. These systemic privileges predispose community members to timely prenatal care entry and routine check-ups, increasing the likelihood of detecting fetal conditions early [11–13]. Conversely, in lower-SES communities, healthcare may be perceived primarily as a resource for emergencies, reflecting systemic marginalization that deters routine care and has been associated with delays the prenatal detection of fetal conditions, such as CHD [5].

Cultural norms also shape healthcare use. In highly religious communities with strict anti-abortion beliefs, social pressures have discouraged fetal anomaly screening due to the perception that screening inherently leads to pressure to consider abortion [14, 15]. Some women have reported perceiving provider bias toward termination after positive screening results, particularly in cases of fetal conditions expected to result in pregnancy loss, stillbirth, or infant death [16–18]. In addition to local influences, The Fetal Condition Care and Outcomes Framework recognizes the significance of broader social contexts. For example, individuals with fetal conditions often lack local support of others with similar experiences and turn to communities online for provider recommendations and peer support, influencing their perceptions and decisions [19, 20].

#### Healthcare policies & organization factors

Federal and state policies create significant variations in care access and outcomes for individuals with pregnancies complicated by fetal conditions. In some regions, policies support timely and comprehensive care, while in others, restrictive laws may delay interventions, exacerbate emotional distress, and worsen outcomes [21–25]. Recent reports highlight cases where individuals in states like Florida and Texas have been forced to continue pregnancies with fatal fetal conditions [26, 27]. Without comprehensive prenatal care coverage, individuals may also forgo critical screenings or diagnostics, leading to missed or delayed fetal anomaly diagnoses [28, 29].

The organization of perinatal health services significantly affects access to and use of perinatal care [30, 31]. In regions lacking specialized services like high-risk obstetric care or genetic counseling, individuals may struggle to obtain accurate diagnoses or appropriate guidance for care of complex fetal conditions [2]. Provider shortages, particularly in rural areas, further limit access to expert care and up-to-date clinical guidance. Inconsistent care delivery can result, leading to poorer outcomes [32–34]. Even when specialized prenatal care is accessible, limited pediatric resources—such as specialized care for medically complex children and disability support programs—may influence decisions about perinatal care.

#### Environmental and population factors

Environmental factors, such as air and water quality, food accessibility, and neighborhood safety, can influence healthcare utilization for individuals with pregnancies complicated by fetal conditions. Poor air quality has been associated with adverse pregnancy outcomes in low-risk pregnancies, including preterm birth and low birthweight, which may exacerbate existing fetal conditions [35, 36]. Inadequate access to nutritious food has been associated with increased risk of preterm birth and certain fetal anomalies, which may compound challenges in pregnancies affected by fetal conditions [37, 38]. Perceived risks and logistical challenges in unsafe or unstable living environments can delay prenatal care, preventing early detection and potential intervention in severe fetal conditions [39].

### Individual characteristics and health behaviors

#### Biological factors

Key factors such as age, family health history, and individual health status can affect the timing and type of care sought during pregnancy. For example, adolescent mothers often face barriers like stigma, limited knowledge of available resources, and reduced insurance coverage, delaying or limiting access to care [40]. In contrast, individuals over 35 are more likely to seek care early due to heightened awareness of age-related risks such as chromosomal anomalies [40]. Thus, maternal age functions both as a risk factor—indicating greater healthcare needs—and as a facilitator, driving earlier engagement through increased risk perception. Chronic conditions such as diabetes, hypertension, and obesity necessitate more frequent and specialized prenatal care, increasing healthcare engagement [41]. However, these conditions can also limit eligibility for certain fetal interventions. Therefore, maternal health conditions can simultaneously enhance general healthcare engagement while restricting access to specific treatments.

#### Sociodemographic factors

Sociodemographic factors, including an individual’s relationship status, support network, employment, living situation, health beliefs, religion, race, and ethnicity interact with biological and contextual influences to shape healthcare access and utilization patterns. Racial and ethnic disparities in perinatal care utilization are well-documented, with Black women in the U.S. less likely to receive adequate prenatal care than White women, even after controlling for SES [42, 43]. Married individuals are more likely to have insurance and receive timely prenatal care while unmarried individuals often experience delays in care initiation due to limited support and coverage [44, 45]. However, conflicting views on pregnancy continuation or treatment options may cause tension complicating healthcare decisions [7]. Beyond partners, a broader support network—relatives, friends, and community members—plays a vital role by providing childcare, transportation, emotional support, and assistance during perinatal appointments. In fact, insufficient support can disqualify individuals from certain fetal interventions [46].

#### Financing & organization

Individual economic factors such as income, insurance coverage, transportation, and workplace benefits critically influence healthcare utilization for pregnancies complicated by fetal conditions. Higher income consistently correlates with earlier prenatal care initiation and increased use of specialized services, including advanced diagnostic testing [47–49]. A systematic review on social determinants of fetal condition detection found that lower SES was linked to delayed prenatal diagnoses [5]. Individuals with comprehensive private insurance receive more timely and adequate care than those relying on public insurance or who are uninsured [50]. For example, Medicaid enrollees can face administrative hurdles such as proof of pregnancy and income verification, delaying coverage and prenatal care initiation [51]. Stable employment with supportive workplace policies facilitate perinatal care engagement by enabling time off for appointments and procedures. In contrast, individuals with unstable jobs, inflexible work schedules, and limited supportive policies may struggle to prioritize healthcare, especially when financial pressures and limited leave options create competing demands [52].

## Perinatal care quality and health outcomes

Once within the healthcare system, contextual and individual factors, having shaped access and use, now interact with healthcare structures and processes to influence care experiences and outcomes [9]. The Donabedian Model provides a framework for understanding how healthcare quality influences outcomes by linking structures—such as personnel, equipment, and policies—to processes, including diagnostics, patient counseling, perinatal interventions, and care coordination. This model has been adapted to various contexts, including Lorch et al.’s 2021 application to neonatal care, which demonstrated how aligning hospital structures, such as levels of care, with processes improves the quality of care and outcomes for high-risk infants [10]. Expanding on these concepts, the Fetal Condition Care and Outcomes Framework (Fig. 1) and case examples (Fig. 2) examines how social determinants of health, such as socioeconomic status, racialization, and geographic disparities, persist as underlying drivers of outcomes, influencing how care is delivered and experienced.

### Structures of care

The structure of healthcare systems directly shapes the availability and quality of perinatal care for individuals with pregnancies affected by fetal conditions. Key structural elements to providing quality care to patients with fetal conditions include specialized personnel, advanced diagnostic equipment, perinatal interventions (e.g., abortion care, perinatal palliative care, prenatal and neonatal treatments targeting congenital conditions) and specialized neonatal care [53, 54]. Structural components are determined by institutional policies and clinical capabilities.

Fetal care centers demonstrate how integrated healthcare structures can improve outcomes through centralized, multidisciplinary teams, that include maternal-fetal medicine providers, pediatric specialists and surgeons, fetal care nurses, genetic counselors, and psychosocial professionals [54, 55]. This structure has been essential for the development of maternal-fetal surgical interventions, enabling safe and effective procedures through coordinated prenatal evaluation, precise surgical planning and transitions to postpartum/postnatal care [53]. Unfortunately, the benefits of fetal care centers remain inaccessible to many, and without access to these specialized services, patients must rely on standard care approaches which often fail to meet the complex needs of families navigating severe fetal conditions [46, 49, 56–59].

### Processes of care

Processes within the healthcare system determine *how* care is delivered after structural resources are in place [9]. For individuals with fetal conditions, these processes include how healthcare teams conduct diagnostic evaluations, counsel patients and families about available management options, coordinate services across specialties, and provide clinical care. These processes can vary significantly based on institutional policies, clinical expertise, and individual provider perspectives, which shape how care decisions are discussed and how interventions are implemented. For example, counseling about management options—including maternal-fetal surgery, neonatal interventions, abortion care, and perinatal palliative care—is shaped by clinicians’ knowledge, beliefs, and comfort levels [60, 61]. Research also shows that the type of clinician leading counseling sessions affects what information is shared, as some may hesitate to discuss certain options due to perceived legal or institutional constraints, while others may struggle to explain complex interventions or feel uncertain about the effectiveness of specific treatments [62–64]. How clinicians perceive the value of different management approaches can influence whether they present options like perinatal palliative care as meaningful or prioritize more aggressive interventions [64, 65]. Patient perspectives reveal significant variation in the experience of receiving counseling, with some individuals emphasizing the empathy, respect, and clarity of their interactions, while others described feeling overwhelmed by medical jargon, pressured in their decisions, or unsupported in understanding their options [7, 66].

### Outcomes of care

Outcomes for individuals affected by fetal conditions vary widely across pregnancy, neonatal, and maternal health outcome measures, with much of this variability remaining unexplained. Evidence illustrates how even after accounting for individual-level variables such as the severity of the fetal condition, disparities persist [4, 5]. This suggests that unmeasured variables within the Fetal Condition Care and Outcomes Framework, such as the patient’s contextual factors and structural and processes of care, play a significant role in shaping outcomes.

Maternal mental health outcomes following a pregnancy complicated by a fetal condition provide a striking example of the variability observed across outcomes. Mental health conditions—the leading cause of maternal mortality in the United States and the most common pregnancy complication—are four to six times more prevalent in individuals experiencing fetal conditions than in those with low-risk pregnancies [67–69]. Cole et al. reported post-traumatic stress disorder (PTSD) symptoms in 19% and major depressive disorder in 23% of patients with fetal anomalies, with younger age and minority racial/ethnic status identified as key correlates [70]. While this study highlights correlated variables, the underlying drivers of disparities in mental health outcome remain poorly understood.

## Implications and future directions

Despite advancements in care for fetal conditions, substantial gaps remain in understanding and addressing disparities in access, use, quality of care, and outcomes. The Fetal Condition Care and Outcomes Framework provides a critical lens for expanding research to inform policies, systems, and practices that improve health outcomes for this high-risk population in the United States.

Much of the evidence informing this framework comes from studies of the broader pregnant population, as fetal anomalies, while not rare, involve relatively small numbers that complicate comprehensive research on outcomes and their drivers [1]. This challenge is reflected in the lack of data on the interactions between policies and patient outcomes within this population, as well as on how individuals access risk-appropriate perinatal care [10]. Health services research is increasingly concentrated on patients already receiving care at advanced fetal care centers, leaving critical gaps in understanding the barriers that prevent others from being referred or reaching these specialized settings [5, 7]. As a result, we are left with an incomplete picture of the pathways and obstacles that shape care access and outcomes, both before and after these patients enter our healthcare systems (Table 1). Addressing this requires two complementary approaches. Multi-site health services research can provide detailed insights into variability and disparities across diverse care settings, while large population-based datasets such as insurance databases offer the power to study outcome drivers. Although widely used in other care areas, many population-level datasets remain largely untapped for the fetal anomaly population due to the lack of comprehensive linked datasets integrating pregnancy and infant records. Overcoming this challenge requires validating and sharing standardized methods to accurately identify maternal-infant dyads affected by fetal anomalies, ensuring consistency and accuracy across studies. Existing measures of sociodemographic stressors often used with population-based datasets, such as the Social Vulnerability Index, must be validated for use with the fetal anomaly population [7]. Both multi-site and population-level dataset approaches must address the complex interactions outlined in the framework, encompassing contextual, individual, structure-, and process-level factors. Interdisciplinary collaboration across health services, clinical, and health equity expert researchers is critical for refining and sharing these methodologies to advance our understanding of care and outcomes.

**Table 1:** Evidence supporting the fetal condition care and outcomes framework + gaps in evidence.

Framework component	Factors	Examples of evidence specific to fetal anomalies	Examples of gaps in evidence
Contextual Characteristics	Community (local)	Misunderstandings about religious guidelines can hinder care. At the Midwest Fetal Care Center, healthcare professionals collaborated with Islamic scholars to address misconceptions among local Somali women about religious prohibitions on fetal interventions [64]. This partnership led to religious leaders issuing a fatwa clarifying that prenatal testing and interventions are permissible under Islamic law.	How do community health literacy levels influence care-seeking behaviors and perinatal outcomes for people with pregnancies affected by severe fetal conditions?
	Community ( > local)	Providers warn patients against searching for diagnosis information online due to the risk of encountering alarming or unreliable statistics, while encouraging participation in virtual peer support groups. These groups have offered benefits like connection and shared experiences but have also exposed patients to misinformation and distressing stories [13].	What patterns of misinformation about fetal anomaly care emerge in virtual communities, and how do they influence health care decision-making for individuals navigating affected pregnancies?
	Health policies	Growing restrictive abortion policies have forced families with complicated twin pregnancies to leave their home states for selective reduction procedures when fetal conditions make it impossible for one twin to survive, threatening the survival of the other [65, 66].	How do state-level differences in Medicaid waiver programs like TEFRA (Tax Equity and Fiscal Responsibility Act), influence long-term health outcomes for children born with congenital anomalies?
	Healthcare organization	Increased density of sonographers in a state has been associated with increased prenatal diagnosis rate of congenital heart disease [26].	How does the distance to specialized fetal care centers impact access to care and perinatal outcomes in pregnancies with severe fetal conditions, and how do these associations vary across states?
	Environmental	Inadequate access to nutritious food increases the risk of nutritional deficiencies, such as folate deficiency, which contributes to fetal conditions like spina bifida and anencephaly, thereby raising the specialized perinatal healthcare needs of this population [6].	How do neighborhood safety metrics, such as violent crime rates and perceptions of safety, influence the frequency of missed prenatal appointments among individuals with pregnancies requiring increased surveillance due to a fetal anomaly?
	Population		How do regional maternal obesity rates influence the timing of prenatal detection and patterns of intervention strategies for structural fetal anomalies?
Individual Characteristics	Biological	Older maternal age has been associated with increased likelihood of prenatal detection of fetal conditions [67, 68].	How do adolescent individuals experience healthcare systems following a fetal anomaly diagnosis, and how do their care trajectories, interactions with providers, and outcomes differ from those of older patients?
	Sociodemographic	Maternal-fetal surgery to treat fetal myelomeningocele, a common and severe form of spina bifida, may be contraindicated due to underlying maternal health risks [69].	How frequently are individuals deemed ineligible for prenatal interventions due to psychosocial reasons, and how do their subsequent care trajectories and outcomes compare to those of individuals who are eligible but choose not to pursue these interventions?
	Financing and organization	Private insurance has been associated with higher eligibility for fetal interventions [45, 53, 70].	How do differences in insurance-provided maternal benefits, such as paid leave, influence care pathways and outcomes among individuals with pregnancies diagnosed with severe fetal anomalies?
Structures	Healthcare system structures, including personnel, equipment, policies, and availability of specialized care.	Infants who had prenatal surgery for spina bifida have been found to be twice as likely to walk independently compared to those treated after birth^71^ Perinatal palliative care programs provide interdisciplinary support focused on comfort, emotional care, and individualized birth planning for individuals facing life-limiting fetal conditions.^72^	What are the differences in care delivery and outcomes for fetal anomaly cases managed at children’s hospital-based fetal centers compared to adult hospital-based fetal care centers?
Processes	The way care is delivered, including diagnosis, counseling, management, and care coordination.	Clinicians’ comfort with addressing psychosocial factors in counseling, such as the financial implications of care or the long-term impact of raising a medically fragile child, varies, with some viewing these discussions as outside their clinical role and others seeing them as essential to informed decision-making [56].	How does the inclusion or omission of psychosocial discussions in clinician-patient counseling impact patient decision-making processes and outcomes?
Outcomes	Health outcomes resulting from care, such as morbidity, mortality, and patient satisfaction.	One in four patients with fetal anomalies screened positive for depression during pregnancy, with over half experiencing financial hardship, such as difficulty affording food or rent [71].	What are the healthcare costs incurred by individuals experiencing perinatal loss due to a fetal anomaly, including hospitalizations, follow-up care, and mental health services?
